# The feasibility of a brain-computer interface functional electrical stimulation system for the restoration of overground walking after paraplegia

**DOI:** 10.1186/s12984-015-0068-7

**Published:** 2015-09-24

**Authors:** Christine E. King, Po T. Wang, Colin M. McCrimmon, Cathy CY Chou, An H. Do, Zoran Nenadic

**Affiliations:** 1 Department of Neurology, University of California, Los Angeles, CA USA; 2 Department of Biomedical Engineering, University of California, Irvine, CA USA; 3 Department of Physical TherapyUniversity of California, University of California, Orange, CA USA; 4 Department of NeurologyUniversity of California, University of California, Irvine, CA USA; 5 Department of Electrical Engineering and Computer ScienceUniversity of California, University of California, Irvine, CA USA

## Abstract

**Background:**

Direct brain control of overground walking in those with paraplegia due to spinal cord injury (SCI) has not been achieved. Invasive brain-computer interfaces (BCIs) may provide a permanent solution to this problem by directly linking the brain to lower extremity prostheses. To justify the pursuit of such invasive systems, the feasibility of BCI controlled overground walking should first be established in a noninvasive manner. To accomplish this goal, we developed an electroencephalogram (EEG)-based BCI to control a functional electrical stimulation (FES) system for overground walking and assessed its performance in an individual with paraplegia due to SCI.

**Methods:**

An individual with SCI (T6 AIS B) was recruited for the study and was trained to operate an EEG-based BCI system using an attempted walking/idling control strategy. He also underwent muscle reconditioning to facilitate standing and overground walking with a commercial FES system. Subsequently, the BCI and FES systems were integrated and the participant engaged in several real-time walking tests using the BCI-FES system. This was done in both a suspended, off-the-ground condition, and an overground walking condition. BCI states, gyroscope, laser distance meter, and video recording data were used to assess the BCI performance.

**Results:**

During the course of 19 weeks, the participant performed 30 real-time, BCI-FES controlled overground walking tests, and demonstrated the ability to purposefully operate the BCI-FES system by following verbal cues. Based on the comparison between the ground truth and decoded BCI states, he achieved information transfer rates >3 bit/s and correlations >0.9. No adverse events directly related to the study were observed.

**Conclusion:**

This proof-of-concept study demonstrates for the first time that restoring brain-controlled overground walking after paraplegia due to SCI is feasible. Further studies are warranted to establish the generalizability of these results in a population of individuals with paraplegia due to SCI. If this noninvasive system is successfully tested in population studies, the pursuit of permanent, invasive BCI walking prostheses may be justified. In addition, a simplified version of the current system may be explored as a noninvasive neurorehabilitative therapy in those with incomplete motor SCI.

**Electronic supplementary material:**

The online version of this article (doi:10.1186/s12984-015-0068-7) contains supplementary material, which is available to authorized users.

## Introduction

Mobility after paraplegia due to spinal cord injury (SCI) is primarily achieved by substituting the lost function with a wheelchair [1]. However, the sedentary lifestyle associated with excessive wheelchair reliance can lead to medical co-morbidities, such as osteoporosis, heart disease, respiratory illnesses, and pressure ulcers [2]. These conditions contribute to the bulk of SCI-related medical care cost [2]. Therefore, restoration of walking after SCI remains a clinical need of high priority.

Current approaches to restoring ambulation after SCI include the use of robotic exoskeletons [3, 4] and functional electrical stimulation (FES) systems [5, 6]. These devices, however, lack intuitive able-body-like supraspinal control, as they typically rely on manually controlled switches. In addition, these systems cannot exploit the neuroplasticity of residual or spared pathways between the brain and spinal motor pools [7]. Hence, novel means of restoring intuitive, brain-controlled ambulation after SCI are needed. If successful, such novel approaches may drastically reduce SCI-related medical costs and improve quality of life after paraplegia due to SCI.

Spinal cord stimulation has recently emerged as a promising method to restore voluntary lower extremity movements to those with SCI [8, 9]. Brain-computer interfaces (BCIs), which enable intuitive and direct brain control of walking via an external device [10, 11], can be seen as an alternative approach. Surveys indicate that those with paraplegia due to SCI highly prioritize restoration of walking as a way of improving their quality of life [12, 13]. In addition, approximately 60 % of survey participants expressed willingness to undergo implantation of an invasive BCI device to restore ambulation [13]. However, before such a system can be pursued, it is necessary to establish the feasibility of brain-controlled overground ambulation. In this proof-of-concept study, we report on a noninvasive BCI-controlled FES system capable of restoring a basic form of overground walking to an individual with paraplegia due to SCI. The study advances our existing BCI systems from applications such as walking in a virtual reality environment (VRE) [14–16] and walking with a treadmill-suspended robotic orthosis [10] to overground walking [11]. If successfully tested in a population of individuals with SCI, the proposed BCI-FES system may lead to the development of a fully implantable BCI system for restoring ambulation after SCI.

## Methods

### Participant screening

Ethical approval was obtained from the University of California, Irvine Institutional Review Board (Irvine, CA, USA). Candidates were recruited from a population of individuals with chronic T6 – T12 SCI. They underwent several screening procedures to rule out severe spasticity, contractures, restricted range of motion, lower extremity fractures, pressure ulcers, severe osteoporosis, orthostatic hypotension, as well as affirm neuromuscular responsiveness to FES (see Additional file 1 for details). A physically active 26-year-old male with a T6 AIS B SCI, with no motor function in the lower extremities and no sensation below the injury level except for minimally preserved bladder fullness sensation, passed all the screening requirements. He provided informed consent to participate in the study. He also consented to the publication of the biomedical data and media, including photographs and videos (consent to publish was also obtained from every person featured in these photographs and videos).

### Training procedure

The participant underwent BCI training to learn how to ambulate within a VRE using attempted walking and idling (i.e. relaxing) as a control strategy. This procedure also generated an EEG decoding model that was subsequently used in BCI-FES experiments. In addition, since the supraspinal areas underlying human gait can become suppressed after chronic SCI, it has been suggested that motor imagery practice may facilitate their reactivation [17]. Therefore, the purpose of the BCI-VRE training was to also facilitate the reactivation of the brain areas responsible for gait control. Finally, the participant simultaneously underwent FES training to recondition his lower extremity muscles in order to be able to stand and walk overground using a FDA-approved commercial FES system (Parastep I System, Sigmedics, Fairborn, OH).

#### BCI training

Similar to our prior studies [10, 15, 16], the participant first underwent a BCI screening procedure to determine if he could control the BCI in a VRE. Subsequently, he underwent BCI training in order to further master BCI-VRE control. Each BCI screening and training visit entailed the same procedure that began with a 10-min electroencephalogram (EEG) recording. During this period, the participant engaged in 30-s-long alternating epochs of attempted walking and idling while seated in his wheelchair [10, 16]. A detailed description of this procedure is given in Additional file 1.

Based on these data, an EEG decoding model was generated offline using the methods described in [10, 15, 16]. Briefly, the EEG epochs were segmented into 4-s-long trials of “Idle” and “Walk” class, transformed into the frequency domain, and their power spectral densities (PSDs) were integrated from 6 to 40 Hz in 2-Hz bins. These spatio-spectral data were then subjected to dimensionality reduction using classwise principal component analysis (CPCA) [18, 19], and discriminating features were extracted using approximate information discriminant analysis (AIDA) [20]. Note that this feature extraction method is rooted in information theory [21] and has been extensively tested in our prior BCI studies [10, 15, 16, 22, 23]. More formally, one-dimensional (1D) features $f\in \mathbb {R}$ were extracted by:

(1) $$ f = \mathbf{T} \Phi(\mathbf{d}),  $$

where $\mathbf {d}\in \mathbb {R}^{B\times C}$ is a single trial of spatio-spectral data (*B*–number of frequency bins, *C*–number of electrodes), $\Phi :\mathbb {R}^{B\times C}\rightarrow \mathbb {R}^{m}$ is a mapping from the data space to an *m*-dimensional CPCA-subspace, and $\mathbf {T}:\mathbb {R}^{m}\rightarrow \mathbb {R}$ is an AIDA transformation matrix.

A Bayesian classifier was then designed as follows:

(2) $$  f^{\star}\in\left\{{\vphantom{\begin{aligned} &\mathcal{S}_{1},\quad \text{if}\quad P(\mathcal{S}_{1}|\,\,f^{\star})>P(\mathcal{S}_{2}|f^{\star})\\ &\mathcal{S}_{2}, \quad \text{otherwise} \end{aligned}\quad,}}\right. \begin{aligned} &\mathcal{S}_{1},\quad \text{if}\quad P(\mathcal{S}_{1}|\,f^{\star})>P(\mathcal{S}_{2}|\,f^{\star})\\ &\mathcal{S}_{2}, \quad \text{otherwise} \end{aligned}\quad,  $$

where $P(\mathcal {S}_{1}|\,f^{\star })$ and $P(\mathcal {S}_{2}|\,f^{\star })$ are the posterior probabilities of idling and walking classes, respectively, given the observed feature, *f* ^⋆^. They were found using the Bayes rule $P(\mathcal {S}_{i}|\,f^{\star })=p(\,f^{\star }|\mathcal {S}_{i})P(\mathcal {S}_{i})/p(\,f^{\star })$, *i*=1,2, where $p(\,f^{\star }|\mathcal {S}_{i})$ is a conditional probability density function (PDF) evaluated at *f* ^⋆^, $P(\mathcal {S}_{i})$ is the prior probability of the class, $\mathcal {S}_{i}$, and *p*(*f* ^⋆^) is the (unconditional) PDF. To simplify calculations, the conditional PDFs were modeled as Gaussians with equal variances. Note that this rendered the Bayesian classifier (2) linear [24]. The performance of the classifier was evaluated offline through stratified ten-fold cross-validation [25].

**Fig. 1:**
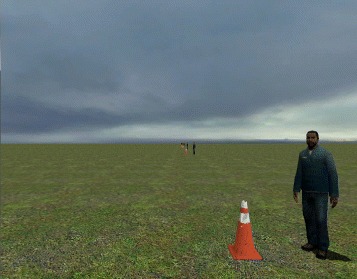
Virtual Reality Environment. A screenshot of the VRE. The traffic cones next to the characters represent designated stops. A full point was given for dwelling at each designated stop for at least 2 s, for a total stop score of 10 points. A fraction of a point was given for dwelling between 0.5 and 2 s (proportionate to the dwelling time) and no point was given for dwelling less than 0.5 s. There was no penalty for dwelling for more than 2 s, but this increased the course completion time. As a benchmark, the course could be completed in ∼205 s with a manually controlled joystick [15, 16]

Each visit continued with online BCI operation, where 0.75-s-long segments of EEG data were wirelessly acquired in real time every 0.25 s using a sliding window approach. The PSDs of the EEG channels were then calculated and integrated in 2 Hz-bins for each of these segments, and used as the input for the EEG decoding model. The posterior probabilities, $P(\mathcal {S}_{1}|\,f^{\star })$ and $P(\mathcal {S}_{2}|\,f^{\star })$, were calculated using the Bayes rule (see above), and were averaged over a 1.5–2.0 s window to minimize false alarms and omissions [10, 15, 16]. Before online BCI operation, the BCI-VRE system was calibrated using a short procedure (see Additional file 1 for details). During each online experiment, the participant performed between one and five goal-oriented, real-time BCI walking tasks. Specifically, he was instructed to utilize attempted walking and idling to control the linear ambulation of an avatar and make sequential stops at ten designated points within the VRE [14–16]. The goal of the task (see Fig. 1) was to walk the avatar at a constant speed and complete the course as quickly as possible, while dwelling at each stop for at least 2 s. The online performances, expressed as the number of successful stops and course completion time, were compared to the results of Monte Carlo simulations to ascertain whether control of the BCI system was purposeful (details in Additional file 1). Note that despite demonstrating purposeful control during the BCI screening process, the participant continued the BCI-VRE training throughout the study. This provided the EEG decoding model for subsequent BCI-FES experiments. It also allowed the participant’s BCI-VRE performance to be tracked over time and the presumed reactivation of the cortical gait areas to occur.

#### FES training

To better understand the FES training procedures, a brief description of the Parastep system’s operation is first provided. Namely, the Parastep achieves ambulation by activating the quadriceps and tibialis anterior muscles. This is accomplished by placing electrode pairs bilaterally over the femoral (immediately proximal to the knee) and deep peroneal (immediately distal to the knee) nerves. Simultaneous bilateral activation of the quadriceps is used to maintain the knee extension necessary for standing, while a front-wheel walker is used for upper body stabilization. A step is achieved with the following sequence: 1. the user performs an anterior-lateral weight shifting maneuver; 2. a brief electrical stimulation is delivered unilaterally to the deep peroneal nerve while the corresponding quadriceps are deactivated, thereby eliciting a triple-flexion reflex of the leg (i.e. combination of foot dorsiflexion, knee flexion, and hip flexion); 3. the user’s leg swings forward due to the anteriorly shifted center of gravity; 4. the quadriceps are reactivated to maintain a standing position. The Parastep system’s adjustable parameters are the step duration (controlled manually by the subject via buttons) and stimulation current for bilateral femoral and deep peroneal nerves. Based on these five parameters, the system generates pre-programmed stimulation sequences for walking movements.

The FDA-approved guidelines for the Parastep system require users to recondition their muscles prior to engaging in FES-mediated walking. This reconditioning also facilitates improved cardiopulmonary endurance. To this end, the participant performed strength and endurance training of the quadriceps using the FES device. Once the participant regained sufficient strength and endurance, and demonstrated the ability to stand using the FES system, the training sessions progressed to FES-assisted overground walking. This included learning the coordination of movements such as weight shifting, front-wheel walker advancement and leg swing, which facilitate FES-mediated walking. A more detailed description of these procedures is provided in Additional file 1. It should be noted that the FES training was also used to empirically determine the stimulation parameters. More specifically, the time necessary to perform the weight shifting, walker advancement, and leg swing determined the step rate. The stimulation amplitude for each femoral nerve was determined as the minimal amount of current necessary to achieve a standing posture. Similarly, the stimulation amplitude for each peroneal nerve was determined by finding the minimal current necessary to elicit an adequate triple-flexion response and step. Note that these parameters were later used in the BCI-FES experiments as described below.

The FES training continued until the participant could walk the length of the overground walking course (3.66 m) without any intervention from the physical therapist. To prevent falls and provide partial body-weight support, FES walking was performed while the participant was mounted in a body-weight support system (ZeroG, Aretech, Ashburn, VA).

### BCI-FES Experiments

The BCI-FES walking experiments were initiated once the participant completed the FES training. This was accomplished by first integrating the BCI and FES systems using a dedicated microcontroller. In addition, the step rate and stimulation amplitudes (as determined above) were pre-programmed into the microcontroller such that the left and right steps cycle automatically. A motion sensor system was then developed and synchronized with the BCI-FES system for the purpose of facilitating the performance assessment. A more detailed description of these steps is provided in Additional file 1. Finally, the EEG decoding model from the most recent BCI training session was loaded into the BCI system. The participant then undertook suspended BCI-FES walking tests followed by overground BCI-FES walking tests.

#### Suspended walking tests

**Fig. 2:**
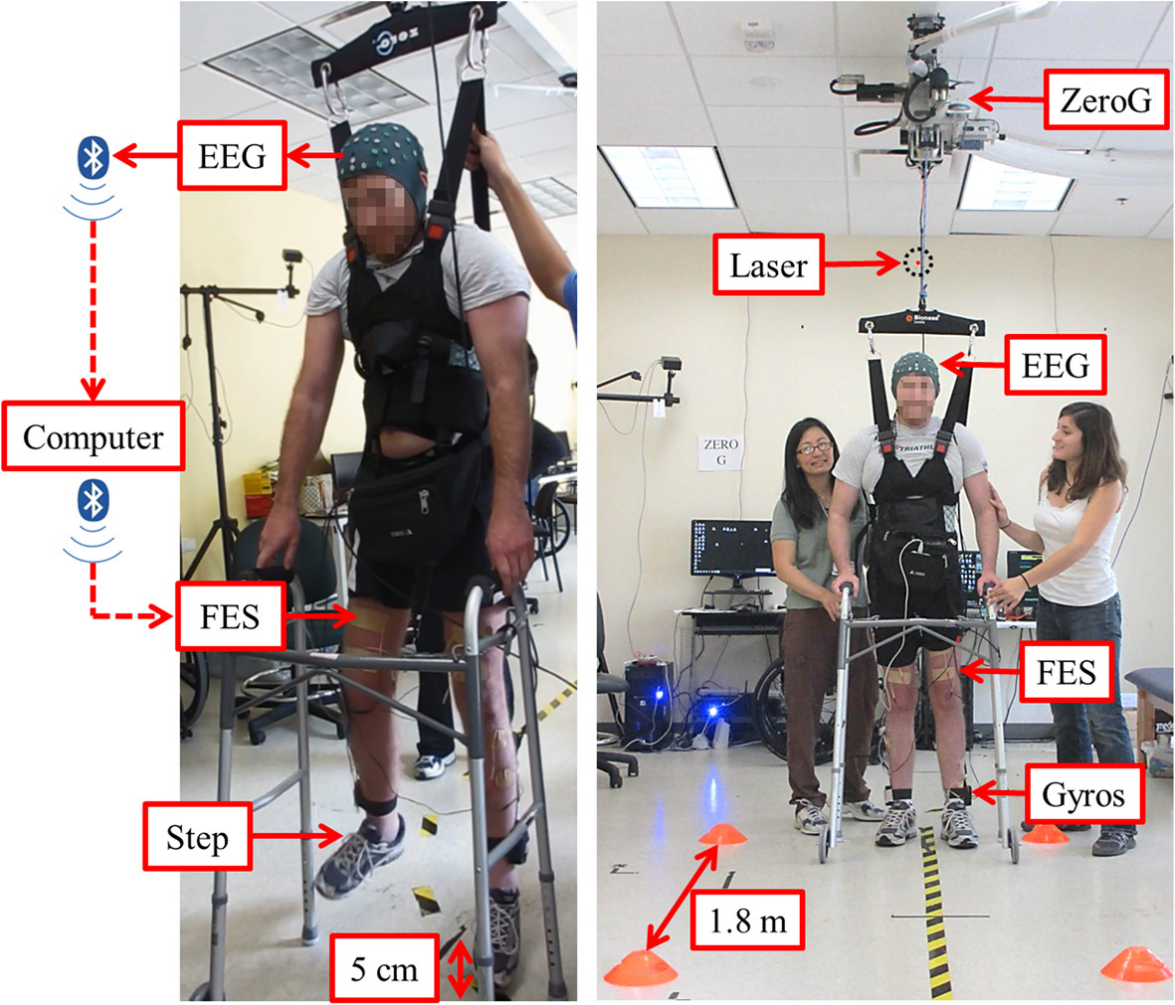
Experimental setup. *Left*: The suspended walking test. In response to “Idle” or “Walk” cues displayed on a computer screen (not shown) the participant modulates his EEG by idling or attempting to walk. EEG is sent wirelessly (via Bluetooth communication protocol) to the computer, which processes the data and wirelessly sends a decision to either “Idle” or “Walk” to a microcontroller. The microcontroller (placed in the belt-pack) drives the FES of the femoral and deep peroneal nerves to perform either FES-mediated standing or walking (in place). *Right*: The overground walking test. In response to verbal cues, the participant performs BCI-FES mediated walking and standing to walk along a linear course and stop at three cones positioned 1.8 m apart. The basic components are: the BCI-FES system, motion sensor system (two gyroscopes and a laser distance meter), and the ZeroG body weight support system to prevent falls. The information flow from EEG to FES is identical to that of the suspended walking test. Note that the participant’s face was scrambled due to privacy concerns

Prior to overground walking, suspended walking tests were performed to establish whether the participant could purposefully operate the BCI-FES system. First, the participant was positioned ∼1 m from a computer screen and suspended using the ZeroG support system so that his feet were ∼5 cm off the ground (see Fig. 2). This allowed the execution of BCI-FES-mediated walking and standing without having to maintain postural stability, perform weight shifting, or advance the front-wheel walker. The participant then followed 30-s-long alternating “Idle” and “Walk” visual computer cues for a total of 180 s with the goal of controlling the standing and walking functions of the BCI-FES system in real time. Finally, the participant’s performance (details below) was assessed using video, BCI state, and motion sensor data.

#### Overground walking tests

For overground walking tests, the participant utilized the system to walk along a 3.66-m-long linear course with three cones positioned 1.83 m apart (Fig. 1). He was instructed to walk and stand at each cone for 10–20 s via verbal cues given by the experimenter. Subsequently, he used an attempted walking strategy to initiate BCI-FES-mediated walking to progress to the next cone. Note that the duration of standing at each cone was randomized to minimize anticipation by the participant. Also note that the ZeroG system was used during these tests to provide partial body-weight support and prevent falls. Overground walking tests were repeated as tolerated by the participant. Video, BCI state, and motion sensor data were recorded to assess the performance during this task.

#### Performance assessment

The subject’s performances in the suspended and overground walking tests were derived based on the video, BCI state, and gyroscope data. Specifically, they were quantified by calculating the cross-correlation and information transfer rate (ITR) between the externally supplied cues and BCI-FES-mediated responses. In the suspended walking tests, the timings of the visual cues were obtained from the BCI computer. In the overground walking tests, the timings of the auditory cues were extracted from the video recordings. In both types of tests, the epochs of BCI-FES mediated responses were extracted from the gyroscope data. Similar to above, purposeful BCI-FES control was ascertained by comparing these cross-correlations to those achieved by Monte Carlo simulations (details in Additional file 1). In addition, the instances of false alarms and omissions were recorded, where a false alarm was defined as the presence of BCI-FES-mediated walking within any intended idling epoch, while an omission was defined as the absence of BCI-FES-mediated walking within any intended walking epoch. Finally, in the overground walking tests, the laser distance meter was used to confirm that the subject ambulated along the course and stopped at the cones.

## Results

### Training

**Fig. 3:**
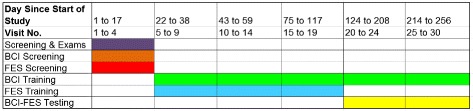
Timeline. Experimental time line of the study

The timeline of the study procedures, including the BCI and FES training, is summarized in Fig. 3. Note that while the participant obtained perfect BCI-VRE control (no omissions or false alarms) after only 11 h of BCI training, the BCI training continued until the end of the study in order to verify that the participant could maintain a high-level of BCI control. In addition, the participant completed the FES training after only 19 FES training sessions, or ∼22.5 h of physical therapy, which is shorter than the Parastep manufacturer’s nominal recommendation of 32 one-hour sessions.

#### BCI training

**Fig. 4:**
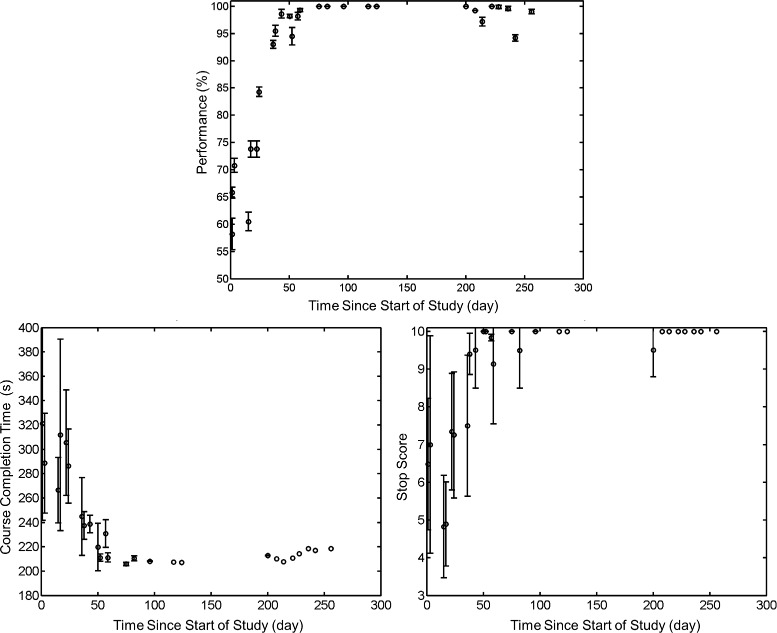
BCI training performances over time. BCI training performances over time. *Top*: Offline performances (%) of the Bayesian classifier (2), as determined through the cross-validation procedure described in the BCI training section. The bar plots represent ± standard deviation (std). *Bottom*: Real-time, online BCI-VRE performances expressed as the course completion time (*left*) and successful stop score (*right*), determined as explained in Fig. 1. The bar plots represent ± std, and data points with no bars indicate that only one VRE session was performed on that day. Note that the participant performed less VRE sessions as the study progressed to make time for more BCI-FES walking sessions

**Fig. 5:**
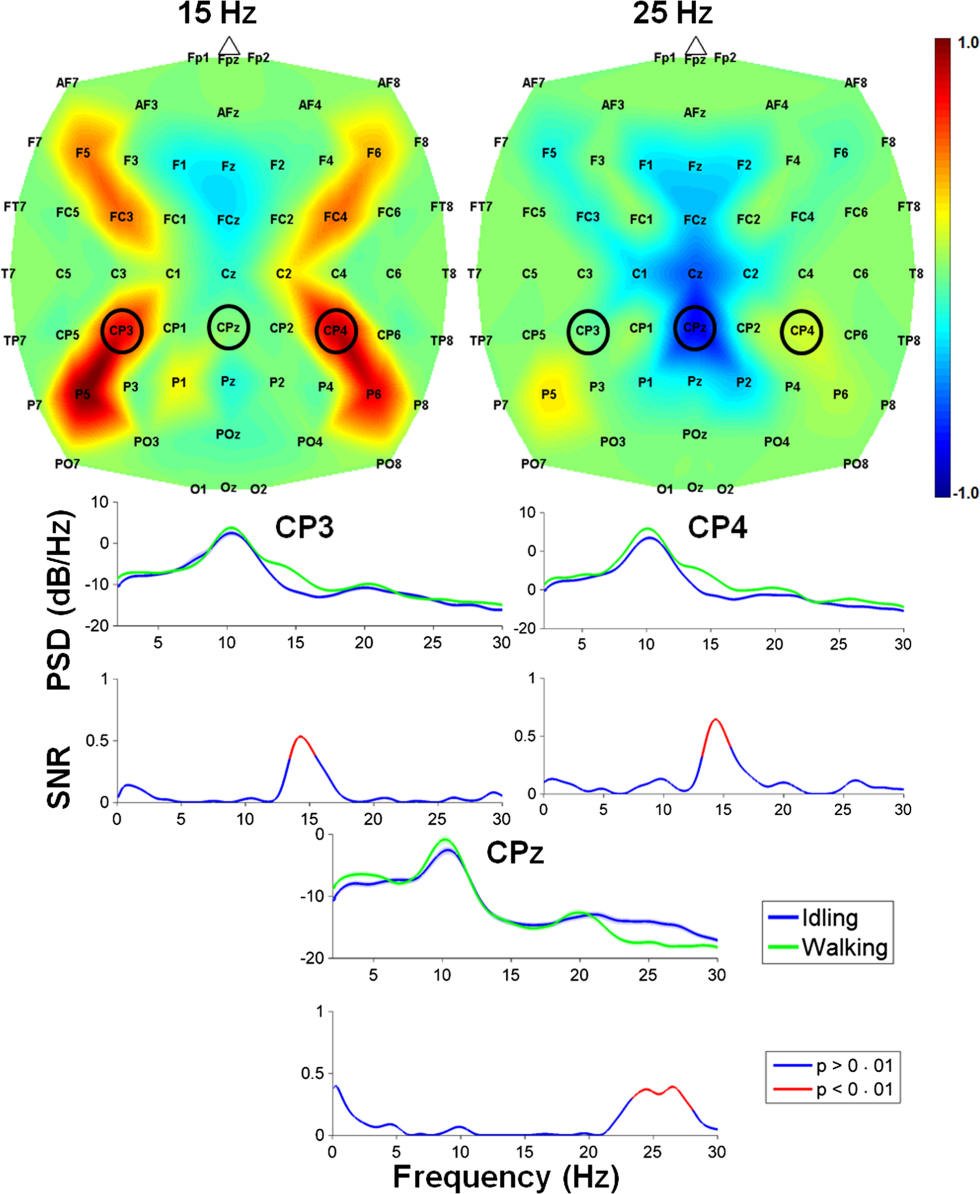
EEG feature extraction maps. *Top*: Feature extraction maps obtained by a combination of CPCA and AIDA for classification of attempted walking versus idling. The spatial distribution of features is shown for the frequency bands centered at 15 Hz and 25 Hz, where the features with values close to ±1 are more important for classification. The maps were generated from data collected during the last visit. *Bottom*: Log power spectral density (PSD) during idling (blue) and walking (green) at electrodes CP3, CPz, and CP4, where shaded regions represent error bars. Underneath the PSD plots are the corresponding signal-to-noise ratio (SNR) plots with significant SNRs (*p* < 0.01) represented by red lines. Note the event-related synchronization (ERS) in the 13–16 Hz range (at CP3 and CP4) and event-related desynchronization (ERD) in the 23–28 Hz range (at CPz)

The performances achieved during the BCI training procedures are shown in Fig. 4. Note that the Bayesian classifier (2) achieved an offline classification accuracy significantly above the chance level (50 %) on the second visit, and a near-perfect classification accuracy by the 15^th^ visit. This translated into a near-perfect level of control during the goal-oriented real-time BCI walking task within the VRE (Additional file 2), which is evident by the decrease in mean course completion time and increase in successful stop score. The EEG decoding models resulted in spatio-spectral features that converged to similar frequencies and brain areas across visits (see Additional file 1). A sample of these features is depicted in Fig. 5, where areas under electrodes CP3, CPz, and CP4 were deemed by the decoding model as important for classification of attempted walking and idling. Note that these areas approximately correspond to the motor and somatosensory cortices. Spectral analysis confirmed the physiological basis of these features, as event-related synchronization (ERS) was observed at CP3 and CP4 in the low- *β* band (13 – 16 Hz), and event-related desynchronization (ERD) was observed at CPz in the high- *β* band (23 – 28 Hz).

#### FES training

The participant typically performed one or two FES training sessions per week. The progression of his FES training is described in detail in Table 1 below. After the visit 19, he demonstrated proper overground walking using the Parastep system. During this training period, it was empirically determined that the participant required 4 s to perform each FES-mediated step. This step rate was programmed into the microcontroller, as explained in the BCI-FES Experiments subsection above. It was also determined that for the suspended walking test, the participant required a nominal stimulation of 120 mA at the femoral nerve, and 50 mA at the deep peroneal nerves. These values were somewhat higher for the overground walking tests, namely, 130 mA for the femoral nerve, and 70 mA and 60 mA for the left and right deep peroneal nerves, respectively. These stimulation parameters were also used for subsequent BCI-FES tests.

**Table 1:** FES training activities across visits. The participant required 19 visits (∼22.5 h of physical therapy) to comfortably walk 3.66 m

Day since	1 – 17	22 – 38	43 – 59	75 – 117
start of study				
Visit no.	1 – 4	5 – 9	10 – 14	15 – 19
	FES screening,	Standing, posture	Weight-bearing support,	Front-wheel walker
	strength training,	and alignment	balance,	management, stepping,
	standing endurance		weight shifting	locomotion

During FES training, the participant experienced a sprain of the left ankle, which was caused by his outside activities. This condition was resolved after one week of rest and periodic icing. The participant also experienced occasional light headedness during his initial attempts of FES-mediated standing and walking. However, this was no longer an issue after the participant progressed to BCI-FES-mediated walking. No other adverse events were observed.

### Suspended walking tests

Once the BCI and FES training were completed, the suspended walking experiments were performed on visits 20 and 21 (Additional file 3). The performance metrics of these tests, including the cross-correlation and lag, number and duration of false alarms and omissions, and the ITR, are presented in Table 2. The participant achieved a very high level of control during this task, as evidenced by cross-correlations as high as 0.957 and ITRs as high as 3.643 bit/s with no false alarms or omissions. The subject’s performance in both of the suspended walking tests was purposeful (*p* < 0.01), according to the criterion outlined in Additional file 1.

**Table 2:** The subject’s performances in the suspended walking tests

Visit no.	*ρ*	Lag (s)	ITR (bit/s)	FA	FA duration (s)	OM
20	0.917	3.00	3.041	1	1.75	0
21	0.957	4.25	3.643	0	0	0
Avg.	0.937	3.630	3.342	0.500	0.880	0

Cross-correlation (*ρ*), lag, and ITR between the cues and the participant’s FES-mediated walking are shown. The number of false alarms (FA), FA duration, and number of omissions (OM) are also shown

### Overground walking tests

**Fig. 6:**
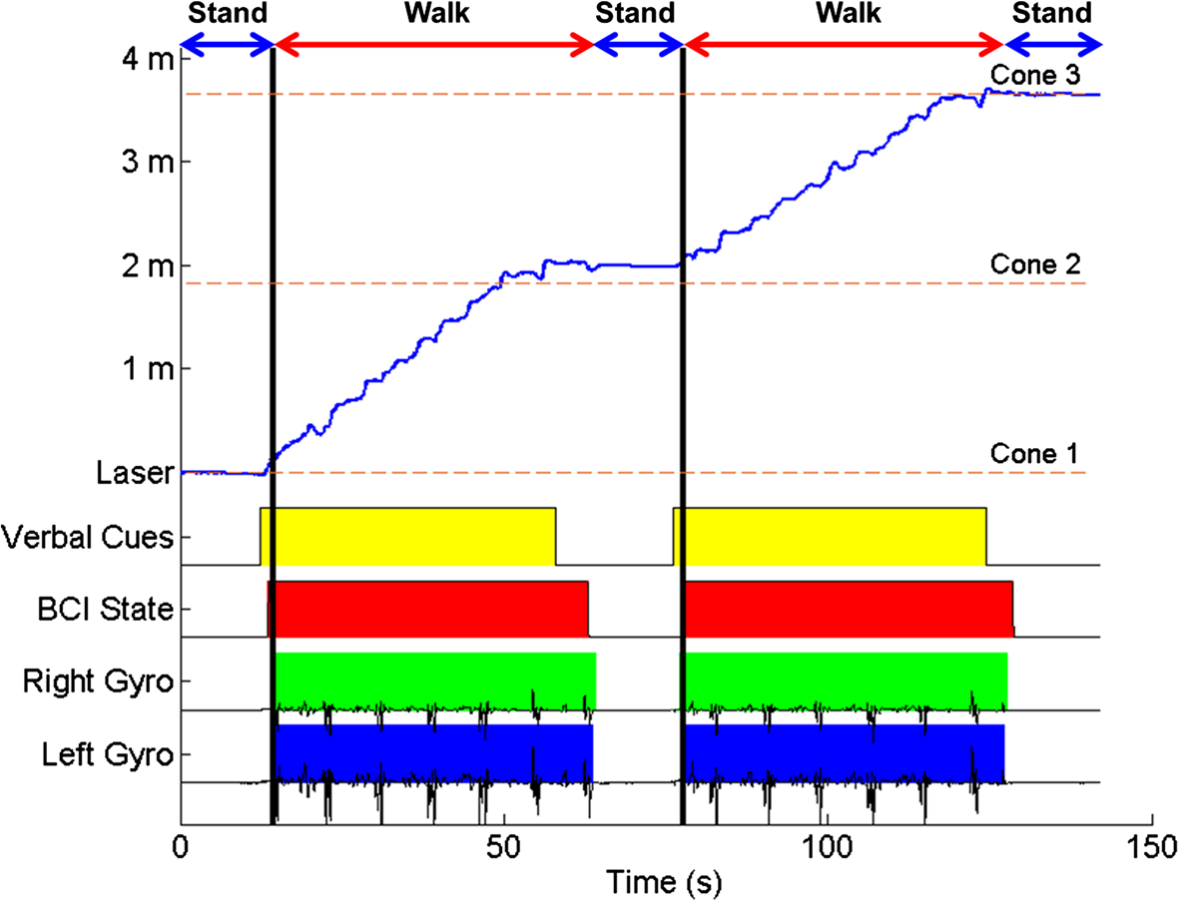
Representative space-state-time plot. The best overground walking test results (data from the 2 ^nd^ test on the 28^th^ visit). The beginning and end of yellow blocks mark the onset of the “Walk” and “Idle” verbal cues, respectively, given by the experimenter. Red blocks represent periods when the BCI system was in the walk state; otherwise, the system is in the idle state. Green and blue blocks represent leg movements recorded by the gyroscopes. The laser signal (blue trace) represents the space-time plot, i.e. the participant’s position within the course as measured by the laser distance meter. Note that there is a delay between the onset of the “Idle” cue and the BCI idle state. This latency includes the time required for the participant’s cognitive processing and EEG to change, as well as the time required for BCI processing. The discrepancy between the onset of the idle state and gyroscope signals is due the fact that transitions from the walk to idle state can be decoded at any time during the pre-programmed 4-s step cycle. For example, if the state transition occurs during an uninterruptible leg swing, the participant will finish the leg swing despite the BCI system being in the idle state (e.g. the first green block). If, on the other hand, the state transition occurs after a leg swing, the leg will be stationary even before the system enters the idle state (e.g. the second green block). Finally, the discrepancy between the gyroscope signals and the distance meter is due to the participant only progressing when the front-wheel walker is advanced, which happens once every 4 s. Hence, all the leg movements prior to walker advancement will be registered by the gyroscope, however, they will not contribute to a position change

Given the promising results above, the participant started the overground walking tests on visit 20 (immediately after the first suspended walking test), and continued these tests until the end of the study (visit 30). In total, 30 overground walking tests were performed over a 19-week period (see Fig. 2). Between one and six overground walking tests were performed on each visit, with each test having an average duration, written in the format mean (standard deviation), of 3.234 (0.743) min. Over time, the participant was able to perform more tests per visit (see Additional file 1). An average cross-correlation between experimenter’s verbal cues and BCI-FES response (i.e. leg movement recorded by gyroscopes, see Fig. 6 and Additional file 4) was 0.775 (0.164) with a 2.861 (4.229) s lag. Note that ∼60 % body-weight support was applied throughout these tests. This value was chosen since it approximates the contribution of the upper body in the total body weight. It was also found to be comfortable for the participant and adequate to prevent falls via the ZeroG’s fall detection algorithm.

The participant had an average of 2.333 (2.039) false alarms (Table 3) and no omissions across all overground walking tests and visits. Comparison to the Monte Carlo simulations also revealed that all 30 overground walking tests were performed with purposeful control (*p* < 0.01). Furthermore, he was able to achieve ITRs similar to the suspended walking tests. In particular, he had an average ITR of 2.298 (0.889) bit/s across all overground walking tests, with a maximum ITR of 3.676 bit/s achieved during the second overground walking test on the 28^th^ visit (see Fig. 6). Finally, no adverse events were observed during BCI-FES-mediated overground walking.

**Table 3:** Cross-correlation (*ρ*) between verbal cues and gyroscope movement, ITR, number of false alarms, and false alarm rate for the 30 overground walking tests performed. Note that the false alarm rates were calculated using the total idling duration. No omissions occurred during any overground walking session

*n*=30	Average	Std. Dev.	Best
*ρ*	0.775	0.164	0.987
Lag (s)	2.861	4.229	2.742
ITR (bit/s)	2.298	0.904	3.676
FA	2.333	2.073	0
FA Rate (FA/s)	0.043	0.039	0

The best session results (on the 28 ^*th*^ visit) are shown in the last column

## Discussion

This study represents the first demonstration of an individual with paraplegia due to SCI purposefully operating a noninvasive BCI-FES system for overground walking in real time. The participant initially operated the system while being completely suspended, and subsequently translated this skill to an overground walking condition. He achieved a high level of control and maintained this level of performance during a 19-week period. These results provide a proof-of-concept for direct brain control of a lower extremity prosthesis to restore basic overground walking after paraplegia due to SCI.

The decoding models for real-time BCI control yielded EEG classification features that were spatially distributed over the motor and somatosensory cortices. A bilaterally-distributed ERS in the low- *β* band (13 – 16 Hz) and a centrally-distributed ERD in the high- *β* band (23 – 28 Hz) were especially prominent. These findings are consistent with prior studies [26, 27], where foot motor imagery resulted in an ERS primarily over the hand representation areas, and an ERD over the foot representation area. These phenomena were observed in both the *μ* (8 – 12 Hz) and *β* (13 – 30 Hz) bands, and are thought to represent an activation of foot representation area with simultaneous inhibition of networks underlying hand movements [26, 27].

The participant achieved and maintained a high level of performance during the BCI-VRE, suspended walking and overground walking tests. In comparison to the suspended walking conditions, there was a notable increase in the false alarm rate during overground walking. This drop in performance could be explained by an increase in EEG noise produced by movements, such as postural stabilization or weight shifting. Nevertheless, the false alarm rate decreased toward the end of the study, presumably due to the participant’s better understanding of the task as well as practice with operating the BCI. Anecdotally, the participant was also able to carry a light conversation during these experiments without interfering with the function of the system. This robustness in real-time control, together with a high-level of performance sustained across months, indicates that BCI-FES mediated restoration of basic walking function after SCI is feasible.

Future studies will focus on testing the function of this system in a population of individuals with SCI. If successfully tested in a larger population, this system may represent a precursor to invasive BCI systems for overground walking. Namely, the cumbersome nature of the current noninvasive system makes its adoption for restoration of overground walking unlikely. This limitation can potentially be addressed by a fully implantable BCI system, which can be envisioned to employ invasively recorded neural signals, such as electrocorticogram or action potentials, as well as implantable spinal cord stimulators [8] or FES systems [28]. Such a fully implantable system would eliminate the need to mount and unmount the equipment, such as an EEG cap, bioamplifier and a computer, thereby making the implantable system more practical and aesthetically appealing. Using an invasive system may also be the only viable approach to deliver cortical stimulation for restoring lower extremity sensation during walking. Nevertheless, the noninvasive system presented here may become a safe test bed to determine which individuals are good candidates to receive these invasive neuroprostheses, once they become available. Furthermore, a simplified future version of the current system may be applied as neurorehabilitative therapy for those with incomplete SCI, whereby residual connections between the brain and spinal motor pools may be strengthened through activity-dependent plasticity mechanisms [29]. In summary, the system reported here represents an important step toward the development of technologies that can restore or improve walking in individuals with paraplegia due to SCI.

## Additional files

Additional file 1 **Appendix.** A supplementary document with additional details, as indicated throughout the body of this report. (PDF 8171 kb)

Additional file 2 **Virtual reality training.** The participant is engaged in using idling and attempted walking to control the linear walking of an avatar in a virtual reality environment. (MP4 10,240 kb)

**Suspended walking test.** The participant is suspended in the air using the ZeroG system. In response to idle/walk cues, the participant utilizes idling/attempted walking to active/de-activate the FES system.

Additional file 4 **Overground walking test.** The participant follows verbal cues from the experimenter, and utilizes attempted walking to perform BCI-FES mediated walking towards the next cone in the course. He then uses idling to stand and dwell until instructed to start walking towards the next cone. (MP4 8878 kb)
