# Supplementary material for: The feasibility of a brain-computer interface functional electrical stimulation system for the restoration of overground walking after paraplegia
Source: J Neuroeng Rehabil. 2015 Sep 24;12:80. doi: 10.1186/s12984-015-0068-7 (PMC4581411; doi:10.1186/s12984-015-0068-7)
Supplement: Supplementary file 1 — Appendix. A supplementary document with additional details, as indicated throughout the body of this report. (PDF 8171 kb) [file 12984_2015_68_MOESM1_ESM.pdf]

Appendix: The Feasibility of a Brain-Computer Interface Functional Electrical Stimulation System for the Restoration of Overground Walking after Paraplegia

The following sections will provide additional details on the methods and results of the study.

1 Methods

1.1 Inclusion/Exclusion Criteria

Individuals with paraplegia due to spinal cord injury (SCI) were recruited according to the following inclusion criteria: age over 18; SCI level between T6 and T12; complete motor paraplegia (AIS A or B) or severe paraparesis (AIS C) due to SCI, resulting in the inability to walk; stable vertebral column to allow upright position; able to provide informed consent prior to the initiation of study; and able to provide transportation to the experimental site. The exclusion criteria were as follows: osteoporosis; prior or current lower extremity fractures; inability to tolerate functional electrical stimulation (FES); inability to control the electroencephalogram (EEG)-based brain-computer interface (BCI) system; presence of any electronic implant (e.g. pacemaker); presence of pressure ulcers; frequent autonomic dysreflexia; orthostatic hypotension; poor truncal control; severe spasticity or contractures; orthopedic malformations that may prevent proper use of the FES system; any neuromuscular disease; cauda equina syndrome; or pregnancy.

To ascertain compliance with the above criteria, candidates underwent several screening and imaging exams. More specifically, these procedures were performed to ensure the participants' ability to safely tolerate the FES and weight bearing during overground walking. In addition, a BCI system integrated with a virtual reality environment (VRE) [1–3] was used for BCI screening in order to ensure that participants could operate the BCI system in real time.

1.2 Screening and Imaging

The candidates were first subjected to neurological and orthopedic exams. Subsequently, a dual energy X-ray absorptiometry (DEXA) scan of the entire body was performed to rule out the presence of severe osteoporosis. In addition, the X-rays of hips, femurs, knees, tibias, fibulas, and ankles, were obtained to rule out fractures and/or lower extremity deformities. Candidates were excluded from the study if they exhibited severe osteoporosis or lower extremity stress fractures.

To rule out orthostatic hypotension, a tilt-table exam was performed using standard clinical practices. Within 5 min of gradual or immediate elevation to 90°, participants must not have exhibited symptoms of orthostatic hypotension such as: a drop in systolic blood pressure by more than 20 points, a drop in diastolic blood pressure by more than 10 points, a pulse increase of more than 30 beats/min, lightheadedness, presyncope, or syncope.

To determine the candidates' neuromuscular responsiveness to FES and whether it causes any adverse events (e.g. autonomic dysreflexia, pain), a brief test was performed. To this end, the electrodes of a FDA-approved, commercial FES system (Parastep I System, Sigmedics, Fairborn, OH) were placed bilaterally over the quadriceps, tibialis anteriors, and gluteal muscles. The candidates' ability to generate effective, FES-mediated muscle contractions without discomfort or adverse events was observed and assessed.

1.3 BCI Screening and Training

EEG Recording

EEG was recorded by a 64-channel, actively-shielded EEG cap (Medi Factory, Heerlen, The Netherlands) mounted on the participant's head. The electrode impedances were reduced to <10 KΩ using conductive gel and scalp abrasion. The EEG data were acquired by a NeXus-32 bioamplifier (Mind Media, Roermond-Herten, The Netherlands) at a sampling rate of 256 Hz. They were wirelessly transmitted to a BCI computer via a Bluetooth communication protocol, and re-referenced with respect to a common average reference. Due to the limitations of the amplifier's wireless transmission protocol, only 24 channels of data were acquired (see Figure 1). Note that this subset of channels was selected because they cover the motor cortex and other brain areas where information pertinent to attempted walking is likely to be located. Also note that these channels are less prone to motion and electromyogram (EMG) artifacts.

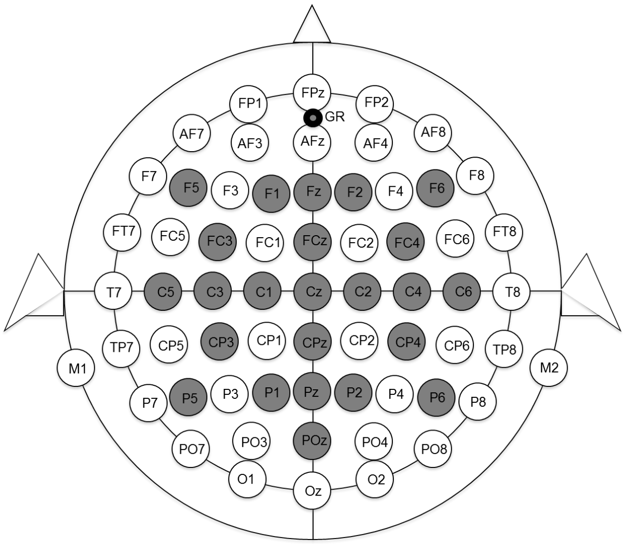

Figure 1: The schematic of a 64-channel EEG cap with electrodes arranged according to the 10–10 International Standard. The subset of channels used in the study is shown in gray.

While seated in a wheelchair, the participant engaged in 30-s-long alternating epochs of attempted walking and idling (relaxation) as guided by textual computerized cues (see Figure 2). EEG data underlying attempted walking and idling epochs were then labeled by an auxiliary signal generated using a secondary data acquisition system (MP150, Biopac, Goleta, CA). NeXus-32 and MP150 systems were synchronized using a common pulse train. The participant was instructed to remain still during the entire procedure, and his movement was monitored by the experimenter. If overt movements were observed, the entire training procedure was repeated.

Calibration

Similar to our previous studies [2–4], the real-time BCI system was implemented as a binary state machine (see Fig. 3), which is governed by comparing the temporally averaged posterior probabilities $\bar{P}(\mathcal{S}_1|f^\star)$ and $\bar{P}(\mathcal{S}_2|f^\star)$ to state transition thresholds, T_I and T_W. To reduce erroneous state transitions during real-time, online operation, the values of T_I and T_W were determined through a short calibration procedure. Briefly, the BCI was set to run in the online mode (with the VRE turned off) while the participant alternated between idling and attempted walking for ∼5 min, as prompted by verbal cues from the experimenter. The underlying EEG data were analyzed offline as explained in the main text, and the average posterior probabilities were calculated. Initially, the thresholds were set as $T_I = \text{median}\{P(S_2|f^\star \in S_1)\}$ and $T_W = \text{median}\{P(S_2|f^\star \in S_2)\}$. Note that this procedure is likely to yield T_I and T_W such that $T_I < T_W$. Also note that this asymmetric threshold structure reduces the participant's mental workload [2]. Finally, the values of T_I and T_W were fine-tuned in a brief familiarization session.

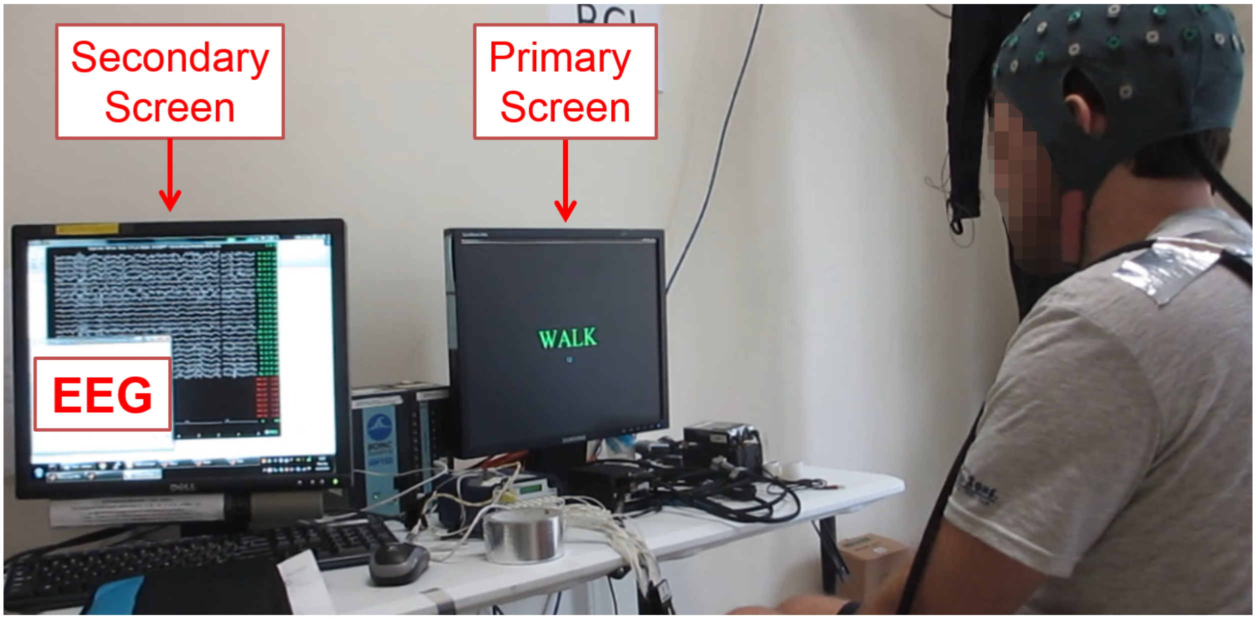

Figure 2: The participant undergoing the BCI training procedure performs attempted walking and idling in response to computer cues shown on the primary screen. Note that the secondary screen (used by the experimenter) was angled such that the participant could not be distracted by it. Also note that the participant's face was scrambled to protect his privacy.

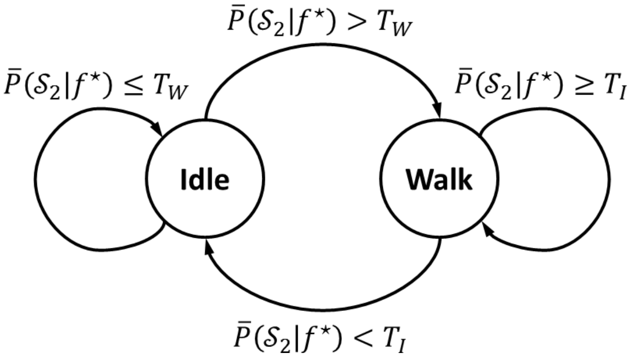

Figure 3: A state machine with the idle and walk states, and state transition rules defined by comparing the average posterior probability of walking, $\bar{P}(\mathcal{S}_2|f^\star)$, to T_I and T_W. For example, if the system is in the idle state and $\bar{P}(\mathcal{S}_2|f^\star) > T_W$, the system transitions to the walk state. Note that since $\bar{P}(\mathcal{S}_1|f^\star) = 1 - \bar{P}(\mathcal{S}_2|f^\star)$, the state transition rules can be expressed as a function of $\bar{P}(\mathcal{S}_2|f^\star)$ only.

Performance Assessment

Since BCI-VRE tasks were performed without externally supplied cues, the ground truth was not known, and therefore the participant's online performances were assessed using the stop score and course completion time [1–3]. To ascertain whether these performances were purposeful, they were compared to the results of Monte Carlo experiments. More specifically, the task was simulated by randomly drawing posterior probabilities and feeding them into the state machine described in the previous section. For this purpose, the threshold values, T_I and T_W, matched those used in the online BCI-VRE tasks. This resulted in a randomly generated sequence of Idle and Walk states, from which the stop score and completion time were calculated. The above procedure was repeated for a total of 1,000 simulation runs, and from these simulated performances, a 2D probability density function (PDF) was estimated using the Parzen window method [5]. Through the subject's online performance point, defined by a successful stop and completion time pair, an iso-PDF contour was drawn. The volume under the PDF outside of this contour was then found by numerical integration, which effectively defines the p-value of the following null hypothesis: "the subject's performance is not different from that of random walk." A performance was deemed purposeful if the empirical p-value of the corresponding Monte Carlo simulation was <0.01.

1.4 FES Training

The participant performed strength and endurance training of his lower extremity muscles, with the goal of being able to bear ∼85% of his body weight in an upright position. These exercises were performed both on site, with the assistance of a physical therapist, and at home. To accomplish this goal, the participant first performed knee extension exercises by having FES applied to his quadriceps. The exercise was repeated with a gradually increasing resistance, which was achieved by attaching ankle weights. Once the participant was able to fully extend the leg with ankle weights equivalent to 10% of his body weight, the strengthening exercise transitioned to FES-mediated standing. These procedures were performed with the help of the physical therapist. To maintain quadriceps strength and endurance, the participant performed daily at-home FES exercises. These consisted of repetitive 10–15-s-long FES-mediated knee extensions while wearing ankle weights. The exercise was terminated after the quadriceps fatigued. In addition, the participant performed FES-mediated foot dorsiflexion exercises for 10–15 s, and repeated this procedure until his tibialis anterior muscles fatigued.

Upon completing the strengthening exercise, the overground gait training began. Its focus was on learning the specific coordination of movements that are necessary for FES-mediated standing and walking. This included mastering the following actions:

1. Standing posture: ability to use FES to stand up from a sitting position, and maintain a proper anterior-posterior/left-right alignment while standing.

2. Weight-bearing support: FES-mediated standing with ∼85% of body weight supported by the legs and ∼15% supported by the arms through the front-wheel walker.

3. Weight transfer: ability to shift body weight in the anterior-posterior direction for advancement and ability to shift body weight to the supporting leg.

4. Stepping: the ability to perform FES-mediated toe off, swing through, and heel strike.

5. Front-wheel walker management: proper placement of the walker during both standing and walking in order to facilitate upper body stability and advancement.

1.5 BCI-FES Integration and Motion Sensor Development

To determine the participant's intention, the wirelessly acquired EEG data (see Section 1.3) were analyzed in real time by the BCI computer, which executed the EEG decoding model from the latest BCI training session. These decisions were then sent to a microcontroller unit (MCU, Arduino, SmartProjects, Turin, Italy) via wireless communication (Bluetooth Mate Silver, Sparkfun Electronics, Boulder, CO). Using digital relays (Relay shield V2.0, Seeed Technology Inc., Shenzhen, China), the MCU was interfaced with the "left step," "right step" and "stand" switches of the Parastep system. Finally, a custom C++ program was uploaded to the MCU to execute an automatic, cyclic stepping pattern in order to induce walking at the

pace determined in the FES training sessions. It should be noted that the MCU only executed these stepping commands when the Walk state was decoded. When the Idle state was decoded, the MCU activated the digital relays responsible for the standing function.

A motion sensor system was developed to facilitate the real-time performance assessment of the BCI-FES system. To accomplish this, two gyroscopes (L3G4200D, STMicroelectronics, Geneva, Switzerland) and a laser distance meter (411D Laser Distance Meter, Fluke Corporation, Everett, WA) with a custom digitizer (LR3 Laser Rangefinder Interface, Porcupine Electronics LLC., Cedar Park, TX), were integrated with a secondary Arduino MCU. An auxiliary signal was generated by this MCU and sent to NeXus-32 in order to synchronize the EEG and motion sensor data for subsequent analyses. The MCU also transmitted gyroscope, laser, and synchronization data to the BCI computer in real time via a Bluetooth communication protocol (Bluetooth Mate Silver). For convenience, the MCUs, NeXus-32, and Parastep's Battery Pack and Stimulator Unit, were placed in a backpack/belt-pack worn by the subject (see Fig. 2 in the main text), while the distance meter was mounted on the trolley of a body-weight support system (ZeroG, Aretech LLC, Ashburn, VA).

1.6 BCI-FES Performance Assessment

Suspended Walking

The performances achieved in suspended walking tests were evaluated by performing cross-correlation and information transfer rate (ITR) analyses on the aligned BCI, motion sensor, and video data. The cross-correlation between the computer cues (representing the ground truth) and BCI-FES-mediated walking/idling was calculated for a range of latencies (lags), and the maximal temporal correlation, ρ, and optimal lag were determined. In addition, false alarm and omission rates, as well as the duration of these errors, were calculated. A false alarm was defined as the initiation of a BCI-mediated walking response within an intended idling epoch. Conversely, an omission was defined as the absence of a BCI-mediated walking response when the intention was to walk. Finally, the ITR (bit/s) was calculated as [6]:

$$\mathrm{ITR} = B\,\mathcal{I}(\mathrm{D},\mathrm{T}) \tag{1}$$

where B is the number of decisions per unit of time (4/s in this study) and $\mathcal{I}(\mathrm{D},\mathrm{T})$ is the mutual information between the true state of the nature, T, as determined by the computer cue, and the state, D, decoded by the computer. Note that the variable T has been lagged according to the value found in the cross-correlation analysis. The mutual information was found as:

$$\mathcal{I}(\mathrm{D},\mathrm{T}) = H(\mathrm{D}) - H(\mathrm{D}\,|\,\mathrm{T}) \tag{2}$$

where $H(\mathrm{D})$ is the entropy of the decoded variable and $H(\mathrm{D}\,|\,\mathrm{T})$ is the conditional entropy of the decoded variable given the true state of the nature. The first term in (2) can be calculated as:

$$H(\mathrm{D}) = -\left[p(\hat{\mathrm{I}})\log_2 p(\hat{\mathrm{I}}) + p(\hat{\mathrm{W}})\log_2 p(\hat{\mathrm{W}})\right]$$

where $p(\hat{\mathrm{I}})$ and $p(\hat{\mathrm{W}})$ are the probabilities of decoding the Idle and Walk states, respectively. Assuming equal prior probabilities, $p(\mathrm{I}) = p(\mathrm{W}) = 0.5$, we have:

$$p(\hat{\mathrm{I}}) = \frac{1}{2}\left[p(\hat{\mathrm{I}}\,|\,\mathrm{I}) + p(\hat{\mathrm{I}}\,|\,\mathrm{W})\right] \quad \text{and} \quad p(\hat{\mathrm{W}}) = \frac{1}{2}\left[p(\hat{\mathrm{W}}\,|\,\mathrm{I}) + p(\hat{\mathrm{W}}\,|\,\mathrm{W})\right]$$

where $p(\hat{\mathrm{W}}\,|\,\mathrm{I})$ is the probability of a false alarm, $p(\hat{\mathrm{I}}\,|\,\mathrm{W})$ is the probability of an omission, and $p(\hat{\mathrm{I}}\,|\,\mathrm{I})$ and $p(\hat{\mathrm{W}}\,|\,\mathrm{W})$ are the probabilities of correctly decoded Idle and Walk states, respectively. The calculations in (2) can be completed after assuming equal priors and realizing that [6]:

$$H(\mathrm{D}|\mathrm{T}) = \frac{1}{2}\left[H(\mathrm{D}\,|\,\mathrm{T}=\mathrm{I}) + H(\mathrm{D}\,|\,\mathrm{T}=\mathrm{W})\right]$$

where the conditional entropy terms are given by:

$$\begin{aligned} H(\mathrm{D}|\mathrm{T}=\mathrm{I}) &= -\left[p(\hat{\mathrm{I}}\,|\,\mathrm{I})\log_2 p(\hat{\mathrm{I}}\,|\,\mathrm{I}) + p(\hat{\mathrm{W}}\,|\,\mathrm{I})\log_2 p(\hat{\mathrm{W}}\,|\,\mathrm{I})\right] \\ H(\mathrm{D}|\mathrm{T}=\mathrm{W}) &= -\left[p(\hat{\mathrm{I}}\,|\,\mathrm{W})\log_2 p(\hat{\mathrm{I}}\,|\,\mathrm{W}) + p(\hat{\mathrm{W}}\,|\,\mathrm{W})\log_2 p(\hat{\mathrm{W}}\,|\,\mathrm{W})\right] \end{aligned}$$

To ascertain whether the subject's performances in the suspended walking tests were purposeful, Monte Carlo simulations were performed. More specifically, the subject's performance in each suspended BCI-FES walking test was compared to the outcomes of 10,000 Monte Carlo simulation runs, and an empirical p-value was calculated. Similar to [4], each Monte Carlo run used the following auto-regressive model:

$$\begin{aligned} &X_{k+1} = \alpha X_k + \beta W_k \quad X_0 \sim U(0,1) \\ &Y_k = h(X_k) \end{aligned} \tag{3}$$

where X_k is a state variable, $W_k \sim U(0,1)$ is uniform white noise, Y_k is the simulated posterior probability (see defined in Section 1.3), and h is a piecewise linear saturation function that ensures $Y_k \in [0,1]$. The coefficients α and β were then determined so that the mean and lag-one correlation coefficient of the sequence $\{Y_k\}$ match those of the posterior probability sequence, $\{P_k\}$, observed in the online tests, i.e.:

$$\begin{aligned} &\alpha = \rho \\ &\alpha\mu + \tfrac{\beta}{2} = \mu \end{aligned} \tag{4}$$

where μ is the mean of $\{P_k\}$ and ρ is the correlation coefficient between P_{k+1} and P_k. Using these coefficients, the posterior probabilities, $\{Y_k\}$, were simulated according to (3) and supplied to the state machine (see Fig. 3) to generate a random sequence of Idle and Walk states. The maximum cross-correlation between this simulated sequence and the computer cue was then calculated. An empirical p-value was defined as a fraction of Monte Carlo trials whose maximum cross-correlation exceeded that achieved by the participant in the suspended walking test. Control was deemed purposeful if the p-value corresponding to the participant's performance was <0.01.

Overground Walking

Similar to suspended walking tests, the subject's performances in the overground walking tests were assessed by performing cross-correlation and ITR analyses between the verbal cues (as recorded by the video data) and the BCI-FES response (estimated based on the gyroscope data). In addition, false alarm and omission rates were calculated. To ascertain whether the subject had purposeful control, 10,000 Monte Carlo simulation runs were generated according to the procedure described above, with verbal cues used as the ground truth. Control was deemed purposeful if the p-value corresponding to the participant's performance was <0.01.

2 Additional Results

2.1 Salient Brain Areas and Frequencies for Classification

The EEG decoding models generated on each visit revealed which spatio-spectral EEG features were salient for distinguishing between idling and walking states. Figures 4 through 9 show the spatial distribution of these features centered at 11 Hz, 15 Hz, 19 Hz, and 25 Hz. The features varied across days, being relatively subtle in the first few visits, and gaining more consistency toward the end of the study. This is especially true for features centered in the low-β (15 Hz) and high-β (25 Hz) bands. In addition, their distribution was mostly confined to the motor and somatosensory brain areas (near electrodes CP3, CPz, and CP4). This is consistent with prior studies [7,8], where in response to motor imagery associated with walking, a bilaterally distributed ERS was observed together with a centrally distributed ERD in both the μ and β-bands. Finally, note that the temporal evolution of the salient feature distribution was concomitant with the improvement of the classification accuracies (Figure 4 in the main text). These may have been caused by reactivation of supraspinal gait control areas [9].

2.2 ERD/ERS and Signal-to-Noise Ratio During BCI Training

The salient spatio-spectral features for classification (Figures 4 through 9) were validated by confirming the presence of EEG power modulation due to attempted walking and idling over the μ (8–12 Hz), low-β (13–20 Hz), and high-β (20–30 Hz) bands (Figure 10). In addition, this figure shows the distribution of the signal-to-noise ratio (SNR) defined as:

$$\mathrm{SNR}(f) = \frac{(\mu_I(f) - \mu_W(f))^2}{0.5\,(\sigma_I^2(f) + \sigma_W^2(f))} \qquad f \in [0,\,30]\,\mathrm{Hz} \tag{5}$$

where $\mu_I(f)$ and $\mu_W(f)$ are the mean power spectra of EEG under idling and walking conditions, respectively, and $\sigma_I^2(f)$ and $\sigma_W^2(f)$ are their respective variances. For each electrode, the PSDs underlying idling and walking were compared across frequencies (0–30 Hz) using a Mann-Whitney U test, in order to identify areas and frequencies with significant SNRs ($p<0.01$). Note that the distribution of significant SNRs (Figure 10) is consistent with the distribution of salient spatio-spectral features in Figure 9.

2.3 BCI-FES Overground Walking Experiments

Figure 11 shows that the number of BCI-FES-mediated overground walking tests performed at each experiment day generally increased over time. The participant started with a single BCI-FES overground walking test (visit 20), and performed as many as six walking tests by the end of the study.

References

[1] Wang, P.T., King, C., Chui, L.A., Nenadic, Z., Do, A.: BCI controlled walking simulator for a BCI driven FES device. In: Proc. RESNA Ann Conf (2010)

[2] Wang, P.T., King, C.E., Chui, L.A., Do, A.H., Nenadic, Z.: Self-paced brain-computer interface control of ambulation in a virtual reality environment. J Neural Eng **9**(5), 056016 (2012)

[3] King, C.E., Wang, P.T., Chui, L.A., Do, A.H., Nenadic, Z.: Operation of a brain-computer interface walking simulator for individuals with spinal cord injury. J NeuroEng Rehabil **10**(77), 1–14 (2013)

[4] Do, A.H., Wang, P.T., King, C.E., Chun, S.N., Nenadic, Z.: Brain-computer interface controlled robotic gait orthosis. J NeuroEng Rehabil **10**(111) (2013)

[5] Duda, R.O., Hart, P.E., Stork, D.G.: Pattern Classification. John Wiley & Sons, New York, NY (2001)

[6] Wang, P.T., King, C.E., Do, A.H., Nenadic, Z.: Pushing the communication speed limit of a noninvasive BCI speller. arXiv preprint **1212.0469** (2012)

[7] Pfurtscheller, G., Muller-Putz, G.R., Schlogl, A., Graimann, B., Scherer, R., Leeb, R., Brunner, C., Keinrath, C., Lee, F., Townsend, G., Vidaurre, C., Neuper, C.: 15 years of bci research at graz university of technology: Current projects. IEEE Transactions on Neural Systems and Rehabilitation Engineering **14**(2), 205–210 (2006)

[8] Pfurtscheller, G., Brunner, C., Schlögl, A., Da Silva, F.L.: Mu rhythm (de) synchronization and EEG single-trial classification of different motor imagery tasks. Neuroimage **31**(1), 153–159 (2006)

[9] Cramer, S.C., Orr, E.L.R., Cohen, M.J., Lacourse, M.G.: Effects of motor imagery training after chronic, complete spinal cord injury. Exp Brain Res **177**(2), 233–242 (2007)

Date	Day	11 Hz	15 Hz	19 Hz	25 Hz
2013-06-25	1	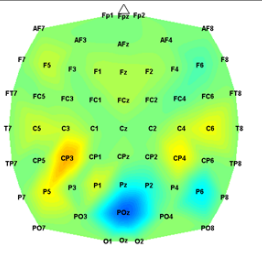	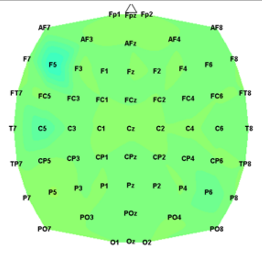	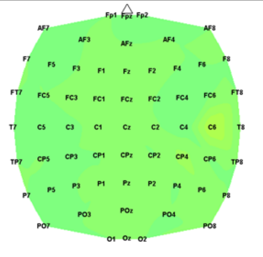	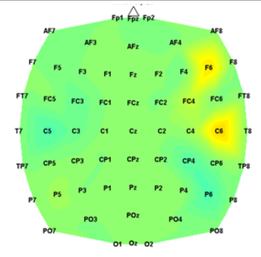
2013-06-27	2	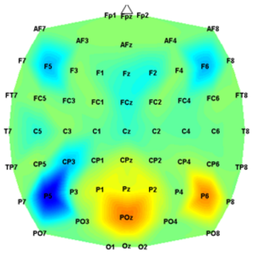	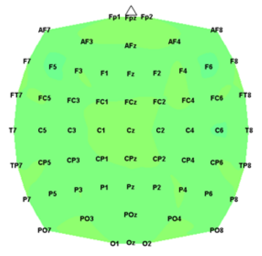	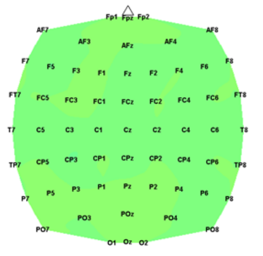	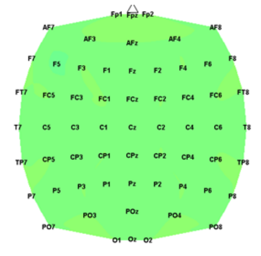
2013-07-09	3	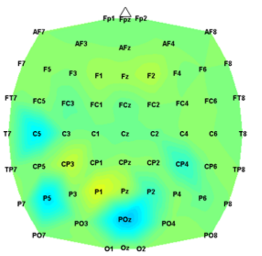	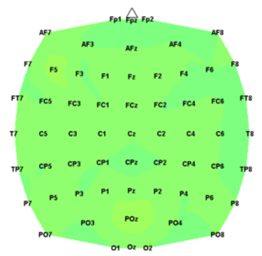	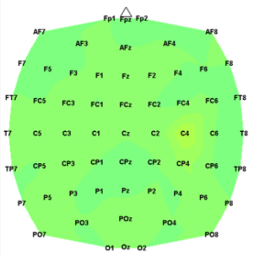	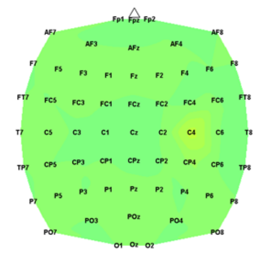
2013-07-11	4	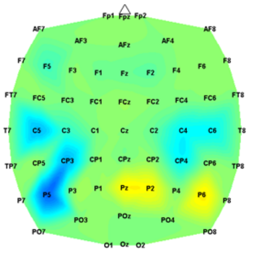	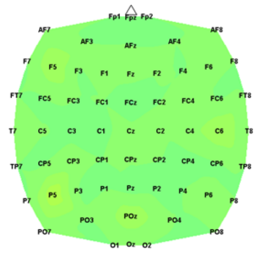	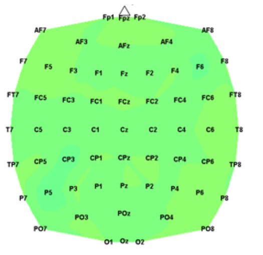	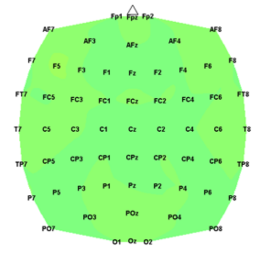
2013-07-11	5	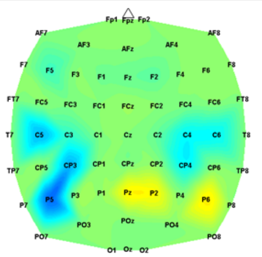	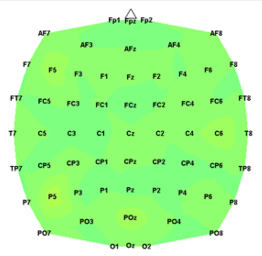	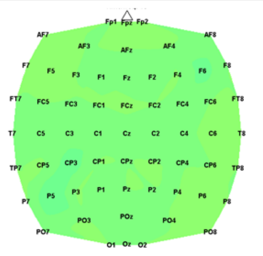	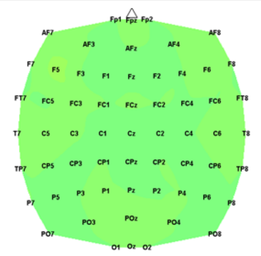

Figure 4: Topographic distribution of the feature extraction maps (Eq. 1 in the main text) over the 11 Hz, 15 Hz, 19 Hz, and 25 Hz frequency bins for visits 1–5. The maps were optimized for the classification of the idle and walk states. Dark red and dark blue colors represent the brain areas that were most important for classification over that particular frequency band.

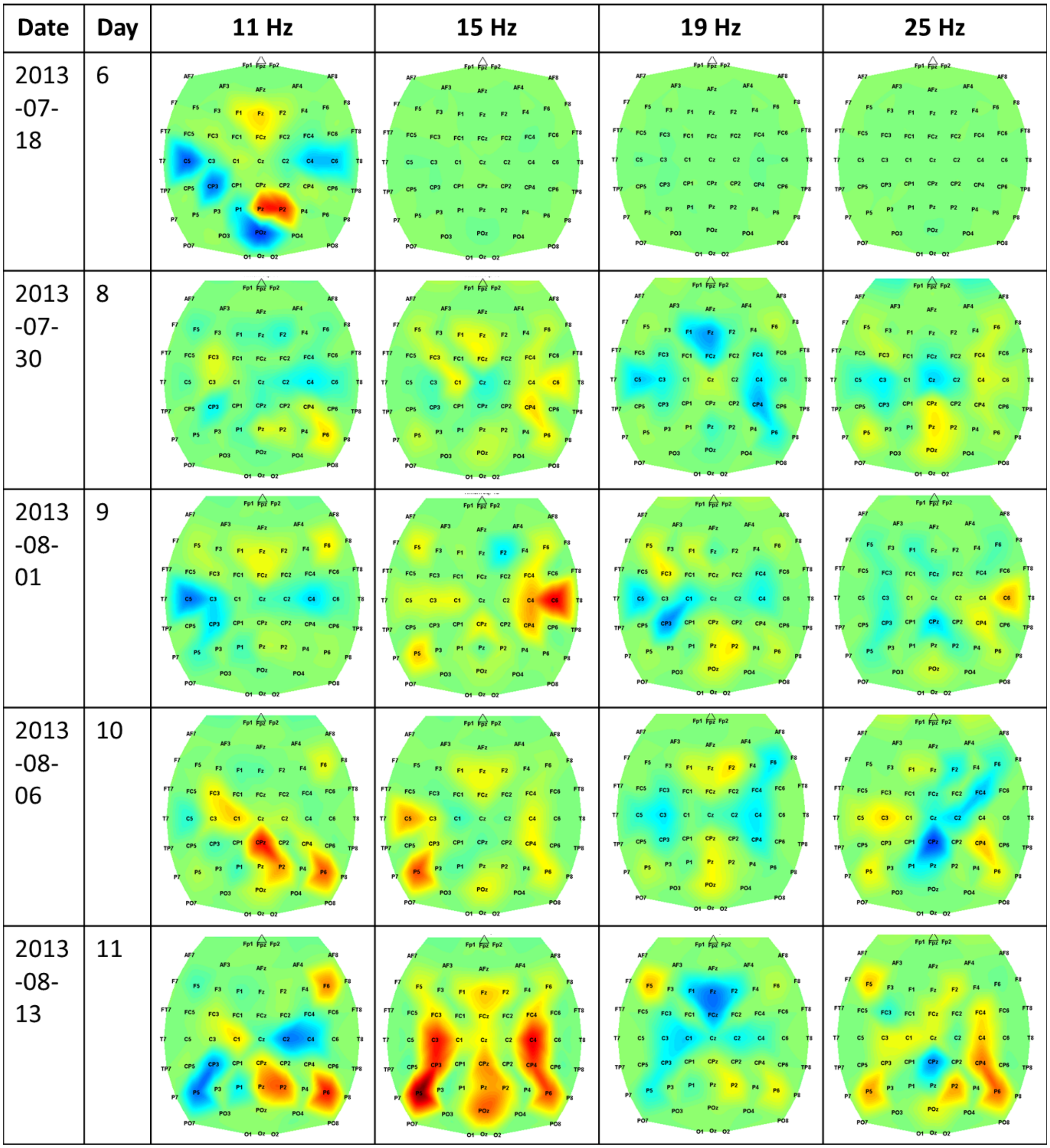

Figure 5: An equivalent figure for visits 6–11.

Date	Day	11 Hz	15 Hz	19 Hz	25 Hz
2013-08-15	12	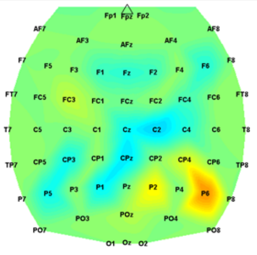	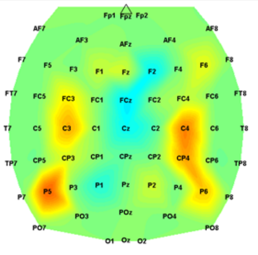	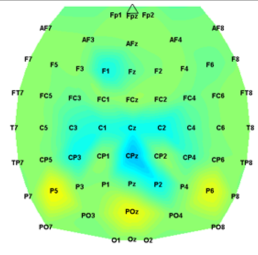	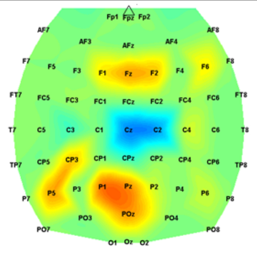
2013-08-20	13	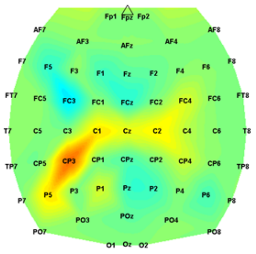	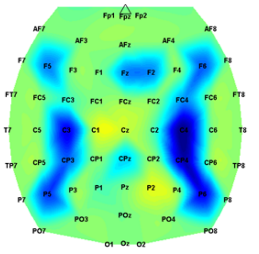	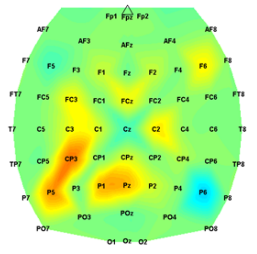	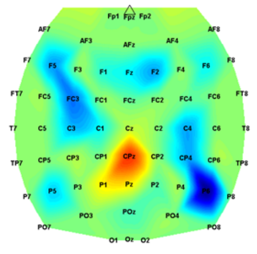
2013-08-22	14	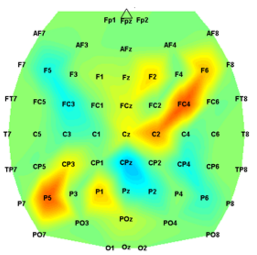	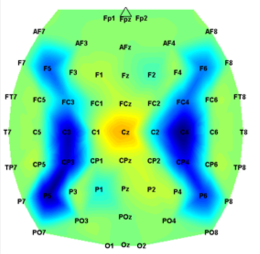	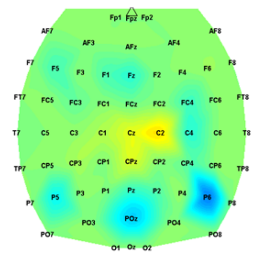	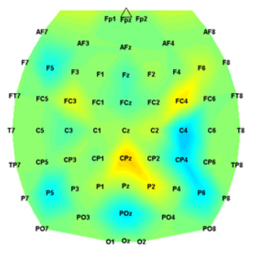
2013-09-07	15	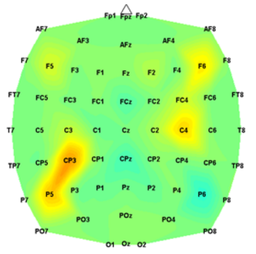	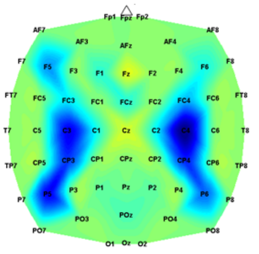	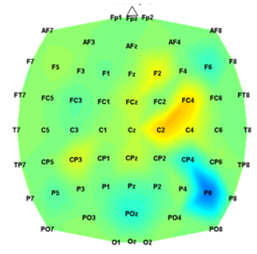	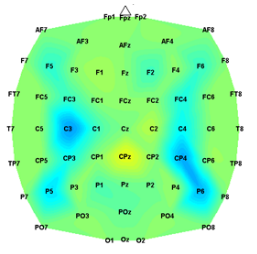
2013-09-14	16	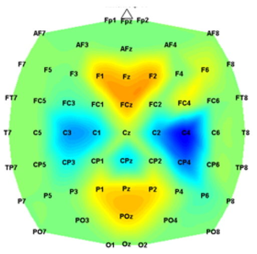	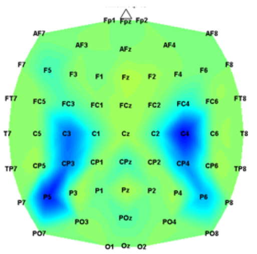	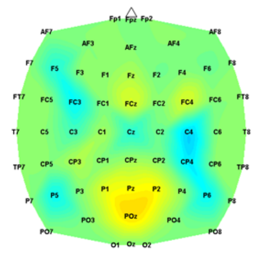	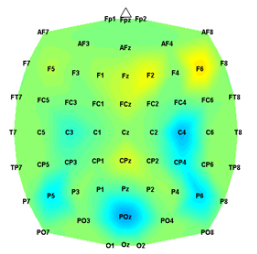

Figure 6: An equivalent figure for visits 12–16.

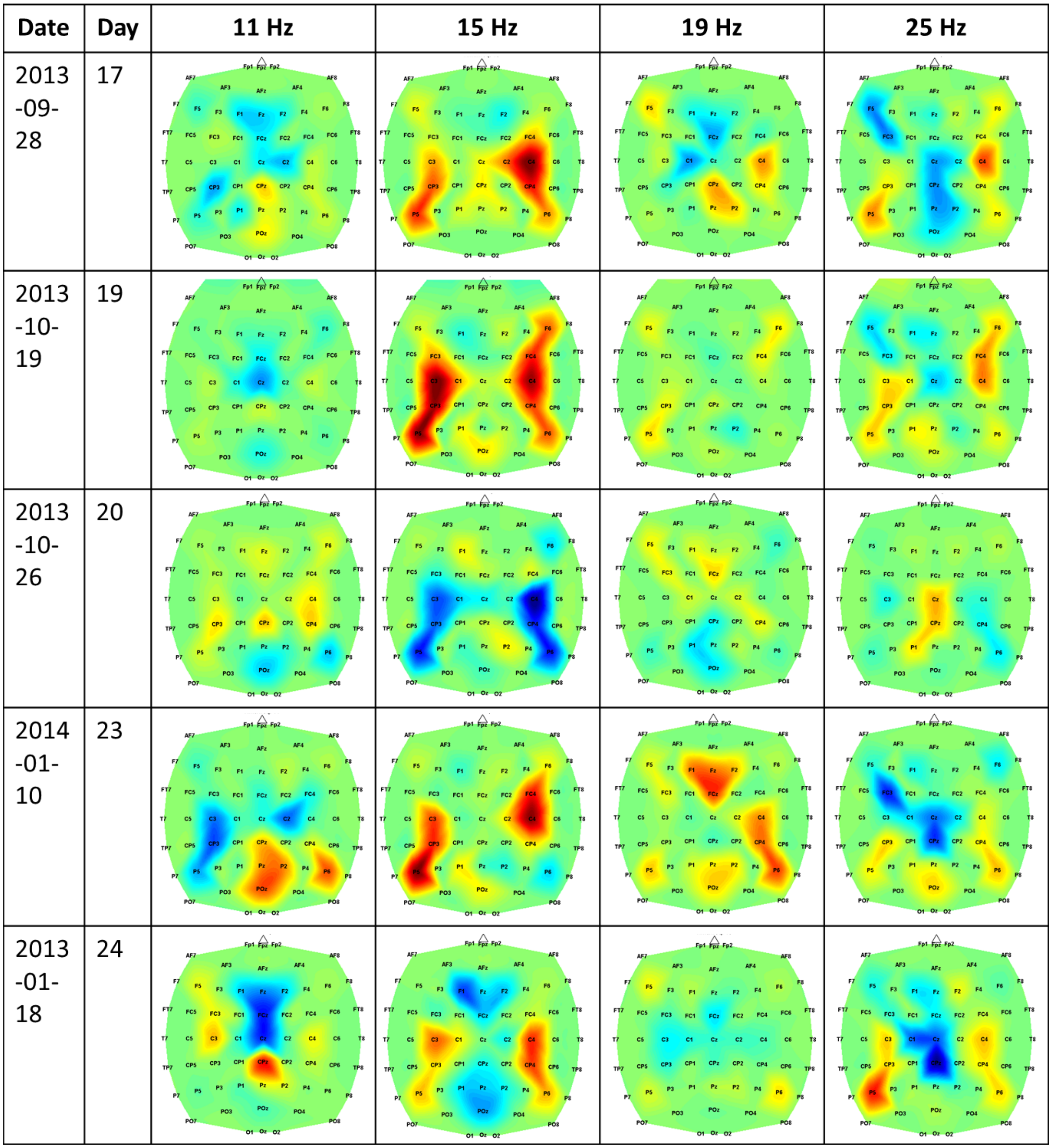

Figure 7: An equivalent figure for visits 17–24.

Date	Day	11 Hz	15 Hz	19 Hz	25 Hz
2014 -01- 24	25	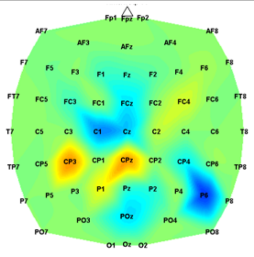	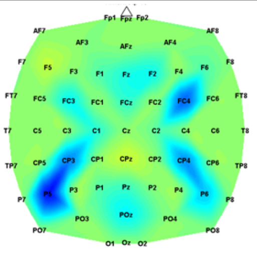	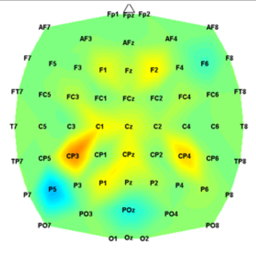	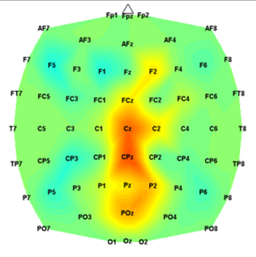
2014 -02- 01	26	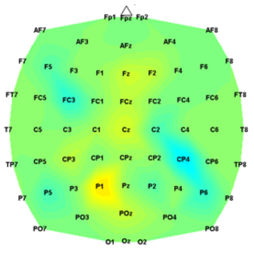	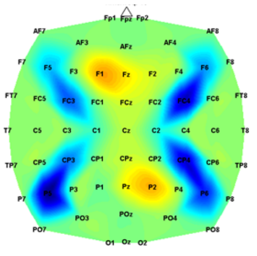	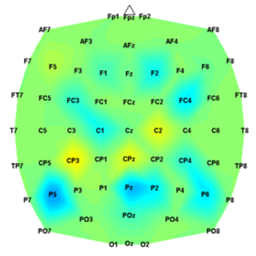	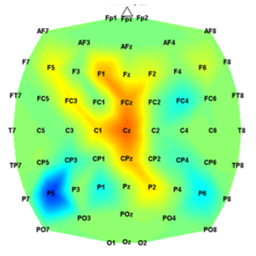
2014 -02- 07	27	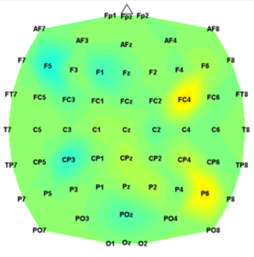	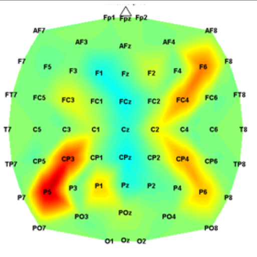	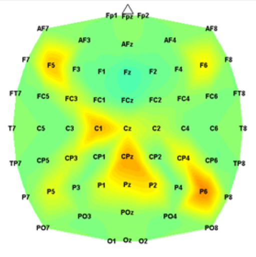	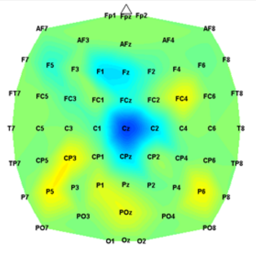
2014 -02- 15	28	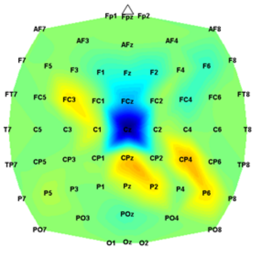	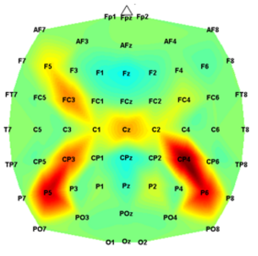	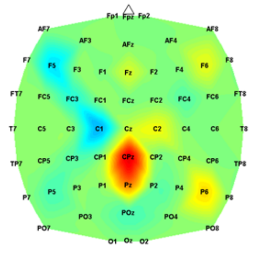	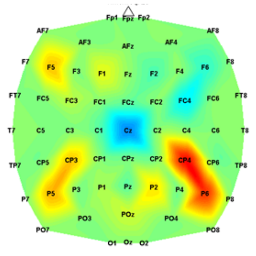
2014 -02- 21	29	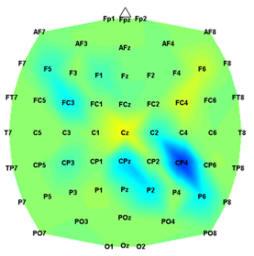	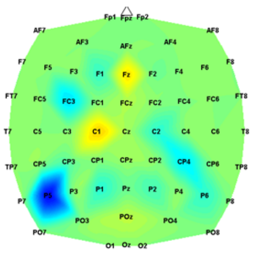	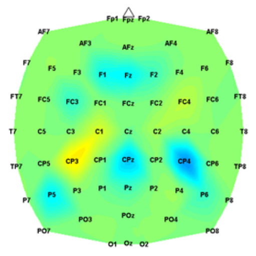	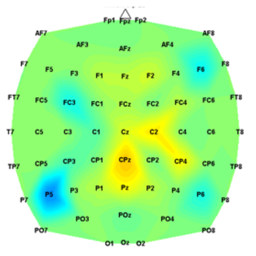

Figure 8: An equivalent figure for visits 25–29.

Date	Day	11 Hz	15 Hz	19 Hz	25 Hz
2014-03-07	30	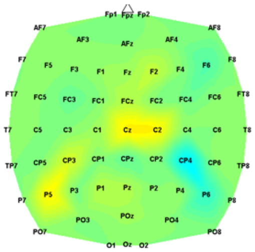	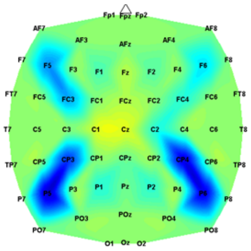	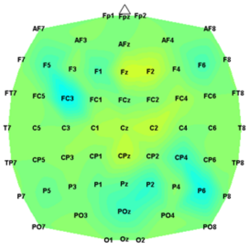	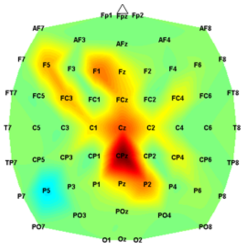

Figure 9: An equivalent figure for visit 30 (last visit).

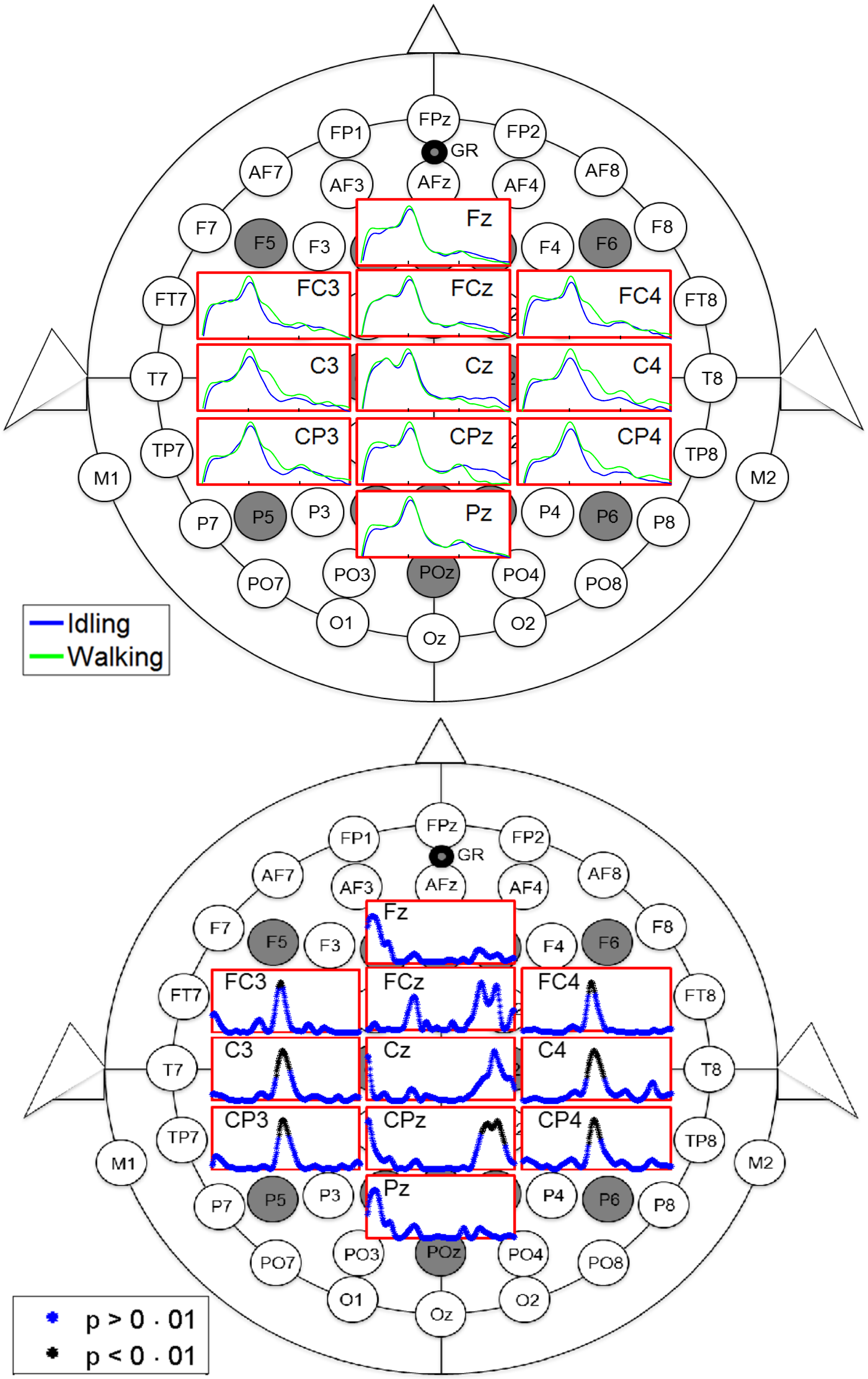

Figure 10: Top: The logarithm of PSDs across frequencies (0–30 Hz, tick marks 10 Hz apart) and electrodes (Fz, FC3, FCz, FC4, C3, Cz, C4, CP3, CPz, CP4, Pz) during BCI operation on the final experimental day. Note the prominent bilateral ERS in the low-β band ($\sim$15 Hz) and central ERD in the high-β band ($\sim$25 Hz) during attempted walking. Bottom: The corresponding distribution of SNR across the same frequencies and electrodes during BCI operation. Note the significant SNR ($p<0.01$) in the low-β band (15 Hz) over electrodes FC3, FC4, C3, C4, CP3, and CP4, and high-β band (25 Hz) over electrode CPz.

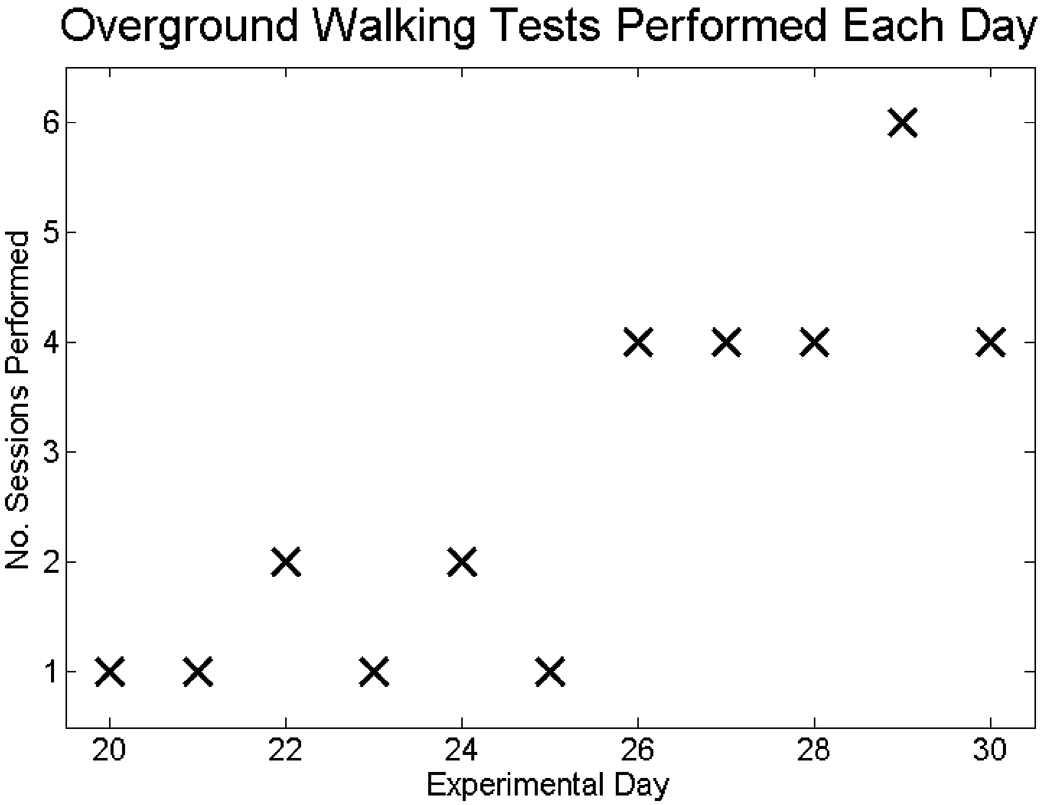

Figure 11: Number of overground walking tests performed on each visit.
